# The tobacco endgame for the Asia Pacific

**DOI:** 10.1111/resp.14465

**Published:** 2023-02-06

**Authors:** Henry M. Marshall, Coral E. Gartner, Kwun M. Fong

**Affiliations:** ^1^ The University of Queensland Thoracic Research Centre Brisbane Queensland Australia; ^2^ The Department of Thoracic Medicine The Prince Charles Hospital Brisbane Queensland Australia; ^3^ NHMRC Centre of Research Excellence on Achieving the Tobacco Endgame, School of Public Health The University of Queensland Brisbane Queensland Australia; ^4^ School of Public Health The University of Queensland Brisbane Queensland Australia

**Keywords:** nicotine, smoking prevalence, tobacco control, tobacco endgame, tobacco industry

**Key points**

- The tobacco epidemic is one of the greatest public health threats, killing over 8 million people annually.
- Despite sustained tobacco control over recent decades, more than 600 million people in the Asia Pacific region smoke.
- Tobacco Endgame strategies aim to rapidly and permanently reduce tobacco use to minimal levels, effectively ending the tobacco epidemic.
- New Zealand has introduced the first national endgame strategy in the region.

Nearly 70 years after the British Doctors Study linked smoking to lung cancer, and cardiorespiratory disease, evidence of smoking's health impacts continues to amass.^1^ The tobacco epidemic is one of the greatest public health threats, killing over 8 million people annually and costing 5.7% of global health expenditure and 1.8% of global gross domestic product. Almost 40% of this cost is borne by low and middle income countries.^2^ Environmental costs are vast.^3^ Annually, six trillion cigarette filters become the second most prevalent plastic pollution and the commonest plastic litter on beaches.^3^

Global treaties support ending the tobacco epidemic.^4^ The 2003 WHO Framework Convention on Tobacco Control (FCTC), the first global health treaty, has been ratified by 182 countries (>90% of global population covered). Tobacco control is integral to The United Nations Sustainable Development Goals. The WHO Global Action Plan for the Prevention and Control of Noncommunicable Diseases (NCD) 2013–2020 targets a 30% reduction in tobacco use by 2025 relative to 2010.

Such efforts reduced smoking from 36.3% to 33.5% in men, and from 7.9% to 6.7% in women between 2009 and 2017.^5^ However, no country has fully implemented all six FCTC/MPOWER measures.^4^ Smoking disproportionately affects people in lower socioeconomic groups, marginalized groups and ethnic minorities. The current paradigm of incremental policy change will see tobacco continue as a leading cause of disease and health inequity for generations.

In contrast, Tobacco Endgame strategies aim to rapidly and permanently reduce tobacco use to minimal levels, effectively ending the tobacco epidemic.^6^ Multiple endgame strategies exist and most countries rely on a suite of policies. Endgame goals vary; some countries aim to eliminate all nicotine products, including smokeless tobacco and Electronic Nicotine Delivery Systems (e.g., Finland^7^), others focus on combustible tobacco products (e.g., New Zealand^8^). Most endgame goals include reaching <5% smoking prevalence between 2025 and 2040.^9^ Multiple policies with endgame potential are described and some are now being implemented. McDaniel identified 16 policies in four focus areas: ‘product’, ‘user’, ‘market/supply’ and ‘larger institutional structures’.^6^ A scoping review found evidence syntheses for eight of these policies, the most researched being very low nicotine content cigarettes, retail restrictions, substitution with non‐combustible products and stringent taxation.^9^

Half the world's population lives in Asia Pacific, corresponding to two WHO Regions—Western Pacific (WPR—37 countries, 1.9 billion people) and South‐East Asia (SEAR—11 countries, 1.97 billion people). These countries are at different stages of the tobacco epidemic.^10^ Some are only just beginning to see prevalence start to decline, while others have reached 10% smoking prevalence after decades of declining prevalence.^11^ Simultaneously, the region dominates tobacco production; 49% of production in 2020 coming from China and India.

Two hundred forty‐one million adults in SEAR and 388 million in WPR smoke. Adult smoking prevalence is projected to fall from 47% in 2000 to 25% by 2025 in SEAR,^12^ although this will remain the highest prevalence of any region. It is projected to fall from 30% in 2000 to 22% by 2025 in WPR, however this falls short of the NCD 30% reduction target.^11^ 14.8 million children aged 13–15 years in SEAR, and 5.7 million children in WPR use tobacco. Smoking prevalence remains low in females, but much higher in males.^10^ Low‐level tobacco use is seen as a market opportunity by the Tobacco Industry and use is rising among women and girls. In eight SEAR and WPR countries, smoking prevalence in girls now exceeds prevalence in women, and in one WPR country more girls smoke than boys.^4^

Sustained tobacco control measures have driven tobacco product diversification, growing markets for alternative products (e.g., ENDS, heated tobacco products and nicotine pouches), flavoured products and increasing concurrent use of multiple products.^13^, ^14^ SEAR has 81% of the world's smokeless tobacco users (240 million), which is 7 times more prevalent among women than smoked tobacco (11.5% and 1.6%, respectively). Tobacco flavourings are unregulated. WPR has the highest prevalence of menthol cigarette use worldwide (15% in 2020), comprising 21%–29% of the market in Japan, the Philippines and Malaysia and 48% in Singapore.^14^ Flavour capsule cigarettes grew substantially in the last decade^15^ and are especially popular in South Korea. Flavourings are important ingredients of ENDS and smokeless tobacco, appealing to youth markets.^16^

Much pioneering tobacco control work occurred in Asia Pacific, such as the first national smoke‐free legislation and advertising bans (Singapore 1970–1971), bans on smokeless product manufacture and sale (Hong Kong 1987) and plain packaging (Australia 2012).^17^ Whilst heavily challenged by the tobacco industry, these policies have inspired others, for example, over 30 countries now have plain packaging laws. The challenges and obstacles to tobacco control in Asia Pacific are similar worldwide^17^ with industry interference ever present, particularly during the COVID‐19 pandemic.^4^, ^17^, ^18^

Asia Pacific is also a pioneer in Tobacco Endgame policy. On 1 January 2023, New Zealand's Smokefree Environments and Regulated Products (Smoked Tobacco) Amendment Act came into force. The Act's clear equity focus aims to redress inequities between Māori and non‐Māori communities,^8^ shifting from individual blame to acknowledging the Tobacco Industry as the source of the problem. It includes a Smokefree Generation law banning tobacco sales to anyone born after 2008; reducing nicotine in cigarettes to very low levels to decrease addictiveness (≤0.8 mg/g tobacco); and reducing access to cigarettes, limiting supply to authorized stores, cutting tobacco outlets by 90%. Companies must also disclose sales, pricing, advertising, sponsorship and ingredients. Funding for health services, campaigns and smoking cessation has increased. This policy package could reduce adult smoking prevalence to 7.3% in 2025 for Māori, and 2.7% for non‐Māori (Figure 1).^19^

**FIGURE 1:**
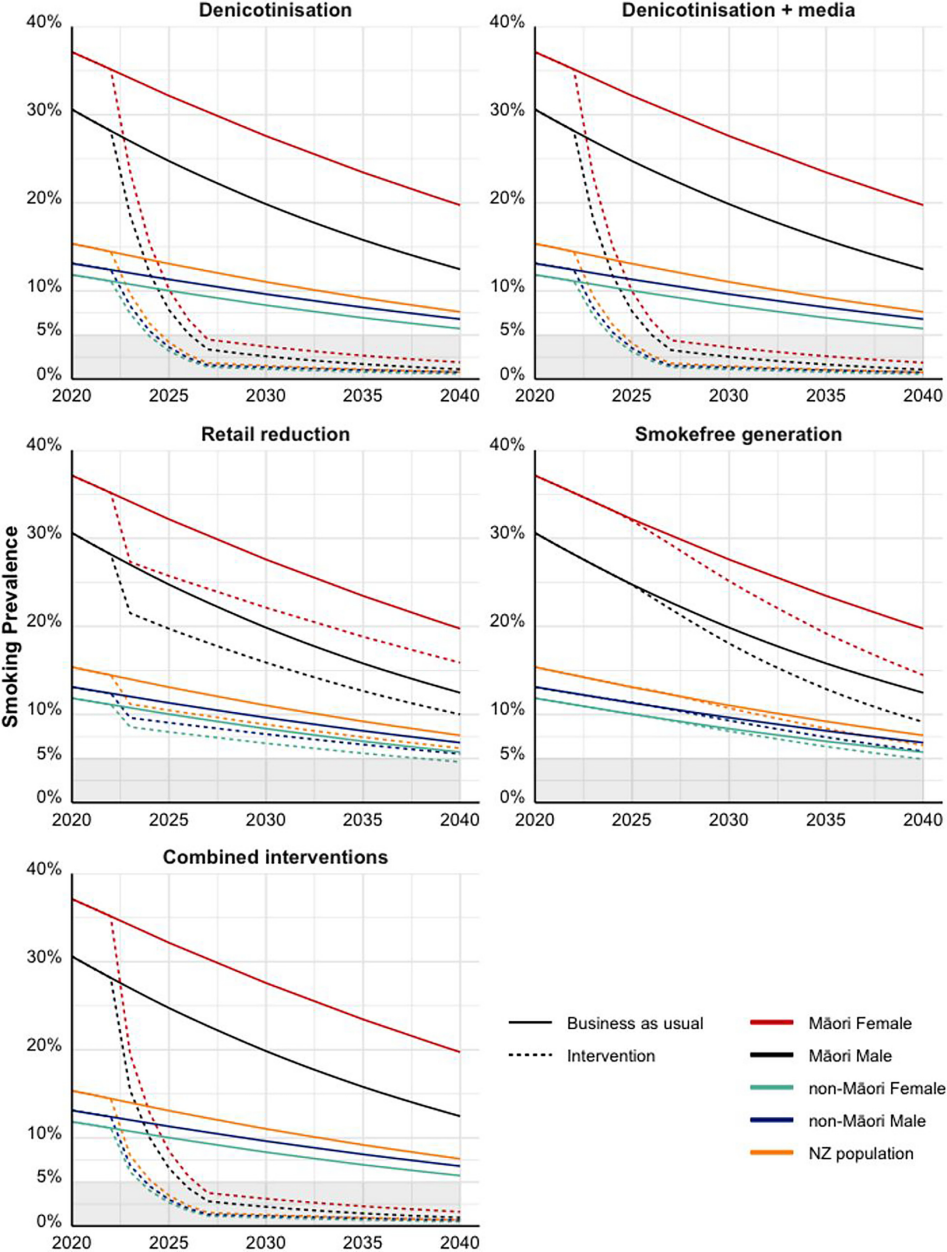
Smoking prevalence (daily, 20+ year population) in Aotearoa New Zealand under business‐as‐usual and endgame interventions (with permission^19^).

In summary, implementation of FCTC/MPOWER tobacco control interventions significantly reduced smoking over the past two decades in the Asia Pacific region. However, millions still smoke and younger generations remain at risk from traditional and novel products. Recognizing the limitations of tobacco control policies, Tobacco Endgame approaches attempt to hasten the end of the tobacco epidemic via bold policies that more directly address key epidemic drivers: tobacco product addictiveness and widespread availability. New Zealand has introduced the first national endgame strategy in the region, setting the standard for other countries to follow.

## CONFLICTS OF INTEREST STATEMENT

Henry M. Marshall is co‐convenor of the Thoracic Society ANZ Tobacco Control Special Interest Group. Coral E. Gartner is President Elect of the Society for Research on Nicotine & Tobacco Oceania Chapter. Coral E. Gartner holds competitive research grants that aim to develop the evidence base for tobacco endgame policies, including an ARC Future Fellowship (FT220100186), an NHMRC Centre of Research Excellence (GNT1198301) and a NHMRC Synergy Grant (GNT2019252). Kwun M. Fong is Past President of the Asian Pacific Society of Respirology.
